# The Resting-State Brain Network Functional Connectivity Changes in Patients With Acute Thyrotoxic Myopathy Based on Independent Component Analysis

**DOI:** 10.3389/fendo.2022.829411

**Published:** 2022-03-24

**Authors:** Yanfang Li, Min Ling, Song Huang, Xinghuan Liang, Yingfen Qin, Zuojie Luo, Jia Zhou

**Affiliations:** ^1^Department of Internal Medicine, Zhuhai Center for Chronic Disease Control, Zhuhai, China; ^2^Department of Radiology, The First Affiliated Hospital of Guangxi Medical University, Nanning, China; ^3^Department of Endocrinology, The First Affiliated Hospital of Guangxi Medical University, Nanning, China

**Keywords:** acute thyrotoxic myopathy, functional magnetic resonance imaging, resting-state brain network, (ICA) independent component analysis, (LFPN) left frontoparietal network, (SMN) sensorimotor network

## Abstract

**Objective:**

The independent component analysis (ICA) was applied to explore the correlation between clinical manifestation and the functional connectivity changes of the sensorimotor network (SMN) and left frontoparietal network (LFPN) in patients with acute thyrotoxic myopathy (ATM), which was expected to provide a functional imaging basis for the exploration of the pathophysiological mechanism of ATM.

**Methods:**

13 ATM patients (ATM) and 12 non-ATM patients (nATM) who met the diagnostic and inclusion criteria were enrolled. Their resting-state brain function images were obtained with resting-state functional magnetic resonance imaging (rs-fMRI). GIFT software was used for independent component analysis to obtain the brain regions with SMN and LFPN changes. The correlation between the functional connectivity of these brain regions and clinical indicators was calculated.

**Results:**

The SMN functional connectivity of ATM patients was increased at the posterior lobe of cerebellum, anterior lobe of cerebellum, right superior temporal gyrus, left cingulate gyrus, left precuneus, and left postcentral gyrus compared with that of nATM patients. However, it was decreased at the occipital lobe, right dorsolateral superior frontal gyrus, paracentral lobule, angular gyrus, and superior parietal gyrus (FDR correction, P<0.05). The LFPN functional connectivity of ATM patients was increased at the posterior lobe of cerebellum, middle temporal gyrus, inferior temporal gyrus, and right cingulate gyrus compared with that of nATM patients; but was decreased at frontal lobe, parahippocampal gyrus, precentral gyrus and postcentral gyrus (FDR correction, P<0.05) Correlation analysis results showed that the enhancement of SMN functional connection at right superior temporal gyrus was significantly negatively correlated with the free thyroxine level, and the decrease of SMN functional connectivity at occipital lobe was significantly positively correlated to the thyroid stimulating hormone level. The SMN and LFPN functional connectivity changes in other brain regions were not found to be significantly correlated with thyroid function parameters.

**Conclusion:**

The bulbar paralysis (such as dysphagia, dysarthria) in ATM patients may be related to the functional connectivity changes of resting-state SMN and LFPN. The fMRI is expected to be one of the objective imaging indicators for the early clinical intervention of ATM patients.

## Introduction

Acute Thyrotoxic Myopathy (ATM), also known as acute hyperthyroid bulbar paralysis, or acute hyperthyroid encephalopathy, is a rare complication of hyperthyreosis involving the muscles innervated by the medulla oblongata in the vital center. The disease has an insidious onset and is easily misdiagnosed and missed. Besides, it progresses rapidly and will lead to high clinical mortality without early clinical intervention (1). Previous studies have not found specific organic changes in the central nervous system in autopsies of ATM patients (2), and medullary paralysis was improved significantly after clinical treatment (3). Therefore, it has been suggested that the symptoms of medullary paralysis in ATM are functional rather than organic changes (4). Given the specificity of the brain, it is not possible to obtain the corresponding tissue specimens at the onset of ATM, and the pathophysiological mechanisms regarding ATM have not been clarified.

In recent years, with the development of neuroimaging, new methods have been proposed for the study on brain function changes caused by thyroid hormone disorders. Resting-state Functional Magnetic Resonance Imaging (rs-fMRI) is a non-invasive brain function imaging technique with a high spatial resolution based on blood oxygen level dependence. Our research team has used degree centrality of rs-fMRI to analyze the differences in brain function networks between ATM patients and healthy controls. The results suggested a correlation between clinical symptoms and altered brain function in the dorsolateral superior frontal gyrus and supplementary motor area (5). The Independent Component Analysis (ICA) is a data processing method for rs-fMRI with high reliability and stability. This method can isolate and analyze multiple resting-state brain networks without prior assumptions (6). It significantly promotes the development of rs-fMRI and provides a neuroimaging basis for the diagnosis of diseases (7). There are no reports of using ICA to study resting-state brain network connection in ATM patients. Sensorimotor Network (SMN) is closely associated with motor initiation, execution, and recovery (8). The left frontoparietal network (LFPN) is mainly involved in language, working memory and motor functions (9, 10) Therefore, in this study, SMN and LFPN were selected to study resting-state brain network functional connectivity changes of patients using ICA. It is expected that this research will provide a functional imaging basis for the study on pathophysiological mechanisms of ATM. It is helpful to further explore the possible pathogenesis of ATM ball paralysis, and enable ATM patients to get timely treatment.

## Materials and Methods

### Subjects

This study selected 13 ATM patients and 12 nATM patients who were diagnosed and treated in the Endocrinology Department of the First Affiliated Hospital of Guangxi Medical University from September 2017 to September 2019. Inclusion criteria: 1) those who meet the diagnostic criteria of hyperthyroidism in the 2016 edition of Guidelines for Diagnosis and Management of Hyperthyroidism and Other Causes of Thyrotoxicosis compiled by American Thyroid Association; 2) those with one symptom of bulbar paralysis (bucking, dysphagia, dyspnea or hoarseness), but without myasthenia gravis, pharyngeal diseases or central nervous system diseases in addition to the general symptoms of hyperthyroidism; (If the patient has muscle strength decline, neostigmine experiment needs to be conducted to exclude myasthenia gravis; if the patient has hoarseness, consultation of otolaryngologist is needed and laryngoscopy should be conducted to exclude laryngopharyngeal diseases; central nervous system diseases need to be excluded by MRI). Exclusion criteria: 1) those with a history of severe cranial trauma; 2) those with neuropsychiatric disorders; 3) those with myasthenia gravis and pharyngeal diseases; 4) those with severe physical diseases such as cirrhosis, cardiopulmonary and renal insufficiency; 5) those with alcohol and drug dependence and abuse; 6) those with other endocrine diseases and autoimmune diseases in addition to hyperthyroidism; 7) pregnant women; 8) those with craniocerebral structure abnormalities confirmed by head MRI.

We collected the sex and age of all subjects. Blood Thyroid hormone levels, such as the Free triiodothyronine, Free thyroxine and Thyroid stimulating hormone, were also collected. On the day the blood data were collected, an MRI scan was performed. Among them, data of ATM patients were collected during their acute course of disease with medulla oblongata symptoms.

This study followed the Declaration of Helsinki and was reviewed and approved by the Ethics Committee of the First Affiliated Hospital of Guangxi Medical University.

### Methods

#### Imaging Equipment and Parameters

The resting-state BOLD-fMRI and 3D T1WI high-resolution structural imaging of all subjects were carried out by Philips 3.0T magnetic resonance scanner and 8-channel phased-array head coil. During the scan, the subjects closed their eyes, relaxed their minds, and stayed awake, with rubber plugs in their ears to reduce noise interference and foam pads on both sides of their heads for fixation.

Scanning parameters of resting-state BOLD-fMRI are as follows: echo time TE=30 ms, field of view FOV=220 mm×220 mm, repetition time TR=2000 ms, matrix=64×62, flip angle=90°, layer number=31, layer thickness=5 mm. Scanning parameters of 3D T1WI high-resolution structural imaging are as follows: echo time TE=3.5 ms, field of view FOV=240 mm×240 mm, matrix=512×512, flip angle=90°, repetition time TR=20 ms, layer number=31, layer thickness=5 mm. All operations were performed by the same highly qualified and experienced radiologist.

#### Data Processing

MRIConvert software was used for format conversion of obtained scanning images, and restplus software was used for image preprocessing. Based on the MATLAB 7.14 (R2013b, Mathworks, Natick, MA, USA) platform, the first 5 time points were removed, and then time correction and realignment were conducted. After that, each subject’s image was aligned with the (Montreal Neurological Institute (MNI) template. Resampling was performed with 3×3×3mm voxels. The Gaussian kernel with full-width at half-maximum of 8mm×8mm×8mm was used for spatial smoothing.

The blind source separation technique-based GIFT (group ICA of fMRI toolbox, http://icatb.sourceforge.net) software was used to perform ICA of the preprocessed data, including data dimensionality reduction, ICA calculation, reconstruction of individual components, and Fisher z-transformation. Components of all subjects were displayed through the Display GUI module of the GIFT software to obtain multiple independent components for each subject and generate independent spatial graphs.The independent components consistent with those reported in the previous study were selected (11).

Functional brain networks are constructed based on brain/neural functional signals (electrical signals, magnetic signals, signals reflecting hemodynamics or metabolism, etc.). Functional connections describe the relationship between functional signals between nodes (which can represent brain functional units at different scales such as neurons, neural clusters, functional brain regions, etc.) in a statistical sense at a given time but do not reflect causal relationships between nodes (12). If the correlation between two brain regions is greater than the standardized threshold value set by statistics, there is a functional connection between them. The functional connection is represented by the t-value. The positive or negative t-value represents the enhanced or weakened information transmission of related functions in different groups. The absolute value of t describes the degree of enhancement or weakening.

The Matlab-based SPM 12 software was used to perform statistical analysis of resting-state brain networks in each group. First, the one-sample t-test (P<0.05, FDR correction) was performed on resting-state brain networks in each group, and the t-value map was displayed and saved as a mask (template) by the xjview software. Then the union of the two sets of the mask was calculated for comparison between groups. The two-sample t-test was used to compare brain regions with different resting-state brain networks in the two groups (P<0.05, FDR correction). Finally, the REST software was used to extract the functional connectivity of brain regions that we were interested in.

#### Statistical Treatment

The general information and clinical data of the two groups of subjects were compared with the statistical analysis software (SPSS 25.0 Inc, Chicago, Illinois), with P<0.05 as the test level. First, the homogeneity of variance on the two sets of general information and functional connectivity was checked. If P>0.05, which indicated that the variance was homogeneous, the differences between groups would be analyzed by the two-sample t-test with the measurement presented as “mean ± standard deviation”. If P<0.05, which indicated that the variance was inhomogeneous, the comparison between groups would be analyzed by the Mann-Whitney U test, with the measurement data presented as “median (25%, 75%)”. In addition, Pearson correlation analysis or Spearman correlation analysis was performed on the clinical indicators and functional connectivity of the two groups.

## Results

### Clinical Information Analysis

A total of 25 subjects were included, 13 cases in the ATM group (4 males and 9 females) and 12 cases in the nATM group (2 males and 10 females). There was no statistical difference in basic information and thyroid function between the two groups. The analysis results are shown in **Table 1**.

**Table 1:** Comparison of basic information and clinical data between the two groups of patients.

Group	The number of cases (n)	Age (year)	Gender (Male/Female)	FT3 (pmol/l)	FT4 (pmol/l)	TSH* (mIU/l)
ATM	13	31.38 ± 7.92	4/9	20.66 ± 13.06	46.16 ± 20.86	0.01 (0.01,0.01)
nATM	12	34.4 ± 11.92	2/10	15.02 ± 11.14	31.06 ± 19.81	0.01 (0.01,0.02)
*t*	\	-0.743	-0.809	1.157	1.852	-1.635
p	\	0.46	0.43	0.26	0.08	0.124

*TSH level showed a non-normal distribution; therefore, the Mann-Whitney U test was used, and the TSH level is presented as median (25%, 75%).

*FT3, Free triiodothyronine in serum; FT4, Free thyroxine in serum; TSH, Thyroid stimulating hormone; ATM, Acute thyrotoxic myopathy; nATM, Non-acute thyrotoxic myopathy hyperthyroidism.

### ICA Analysis Results

**Figure 1:**
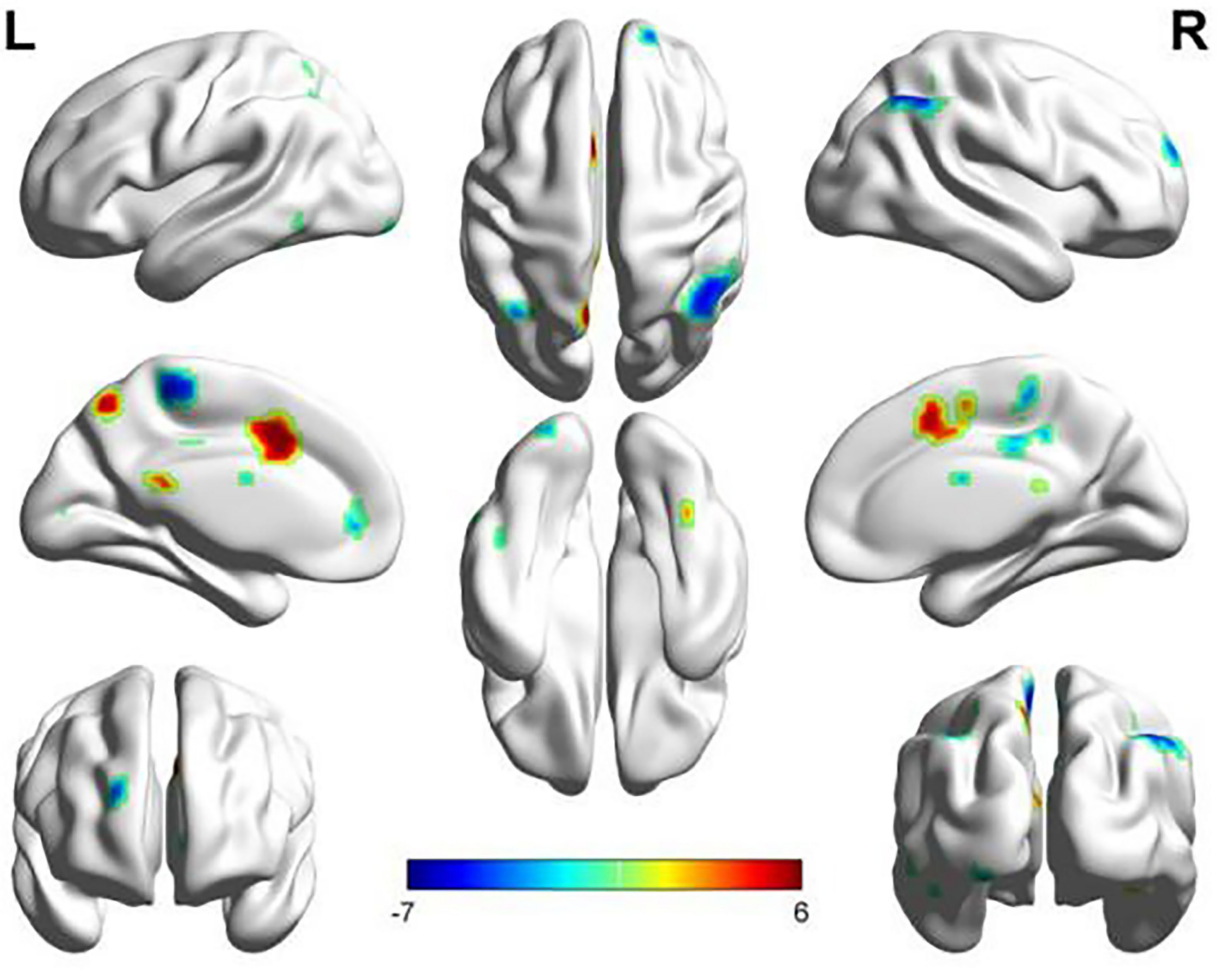
The abnormal brain regions and functional connectivity found in the sensorimotor network in ATM and nATM groups. Red indicates the brain region with enhanced functional connectivity in the ATM group, and blue indicates the brain region with the weakened functional connectivity.

The SMN and LFPN were acquired with GIFT software (13).The SMN functional connection of the ATM group was enhanced in the posterior lobe of cerebellum, anterior lobe of cerebellum, right superior temporal gyrus, left posterior cingulate gyrus, left precuneus, and left postcentral gyrus compared with that of the nATM group; but was weakened in the occipital lobe, right dorsolateral superior frontal gyrus, paracentral lobule, angular gyrus, and superior parietal gyrus (as shown in **Table 2** and **Figure 1**).

**Table 2:** The abnormal brain regions and functional connectivity found in the sensorimotor network in ATM and nATM groups.

Brain region	MNI coordinate			voxel cluster	t value
	X	Y	Z		
Posterior lobe of cerebellum	3	-66	-54	108	5.63
Anterior lobe of cerebellum	21	-48	-27	190	6.56
Right superior temporal gyrus	63	0	0	13	4.34
Left cingulate gyrus	-6	-39	18	10	4.72
Left precuneus	-6	-66	54	55	6.03
Left postcentral gyrus	0	-30	78	18	9.89
Superior/middle/inferior occipital gyrus	-24	-87	3	32	-4.88
Right dorsolateral superior frontal gyrus	24	60	27	12	-4.82
Paracentral lobule	3	-27	42	110	-5.83
Angular gyrus	45	-51	45	133	-5.47
Superior parietal gyrus	-36	-63	51	36	-5.13

MNI, Montreal neurological institute; the statistical results were corrected by FDR, P < 0.05.

**Figure 2:**
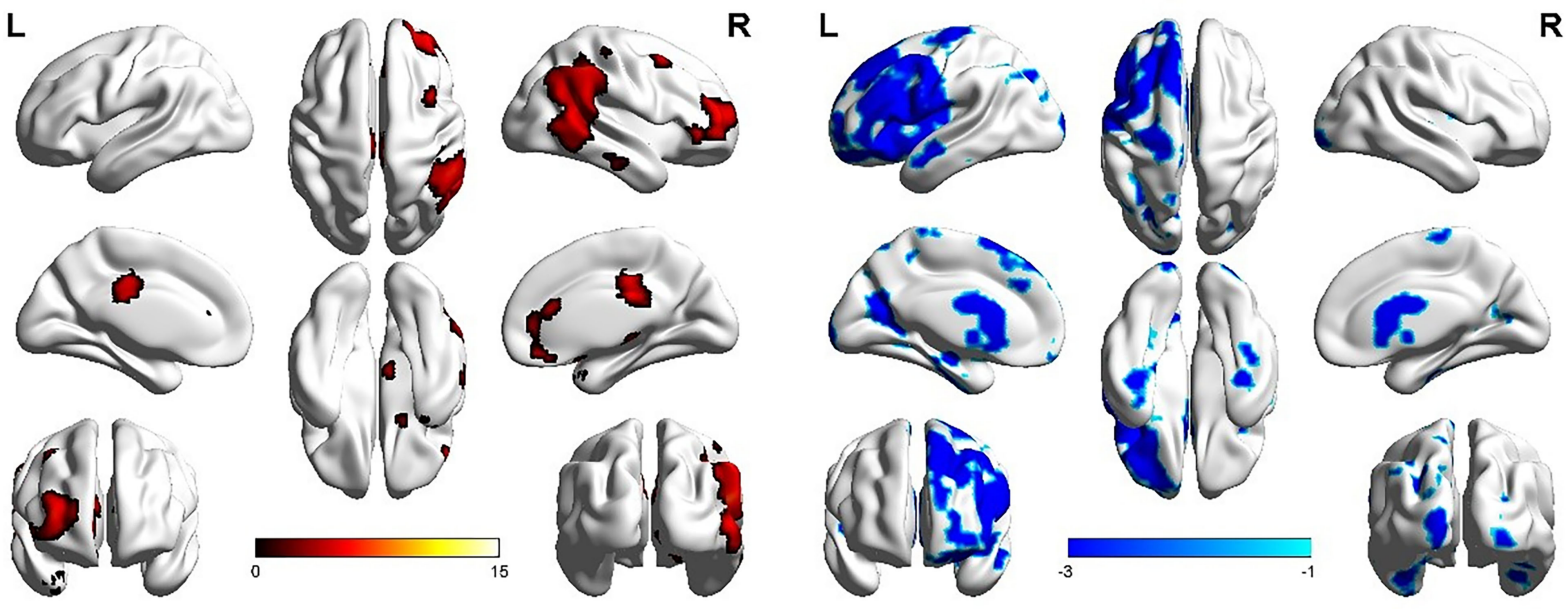
The abnormal brain regions and functional connectivity found in the left frontoparietal network in ATM and nATM groups. Red indicates the brain region with enhanced functional connectivity in the ATM group, and blue indicates the brain region with the weakened functional connectivity.

The LFPN functional connection of the ATM group was enhanced at the posterior lobe of cerebellum, middle temporal gyrus, inferior temporal gyrus, and right cingulate gyrus compared with that of the nATM group and was weakened at the frontal lobe, parahippocampal gyrus, precentral gyrus, and postcentral gyrus (**Figure 2** and **Table 3**).

**Table 3:** The abnormal brain regions and functional connectivity found in the left frontoparietal network in ATM and nATM groups.

Brain region	MNI coordinate			voxel cluster	t value
	X	Y	Z		
Posterior lobe of cerebellum	-51	-48	-48	38	7.36
Middle temporal gyrus	36	6	-33	27	5.77
Inferior temporal gyrus	72	-30	-21	29	4.29
Right cingulate gyrus	3	36	15	76	4.81
Frontal lobe	-36	-42	30	12260	-12.13
Parahippocampal gyrus	36	-21	-30	84	-4.76
Precentral/postcentral gyrus	66	0	21	25	-3.24

MNI, Montreal neurological institute; the statistical results were corrected by FDR, P < 0.05.

### Correlation Analysis

**Figure 3:**
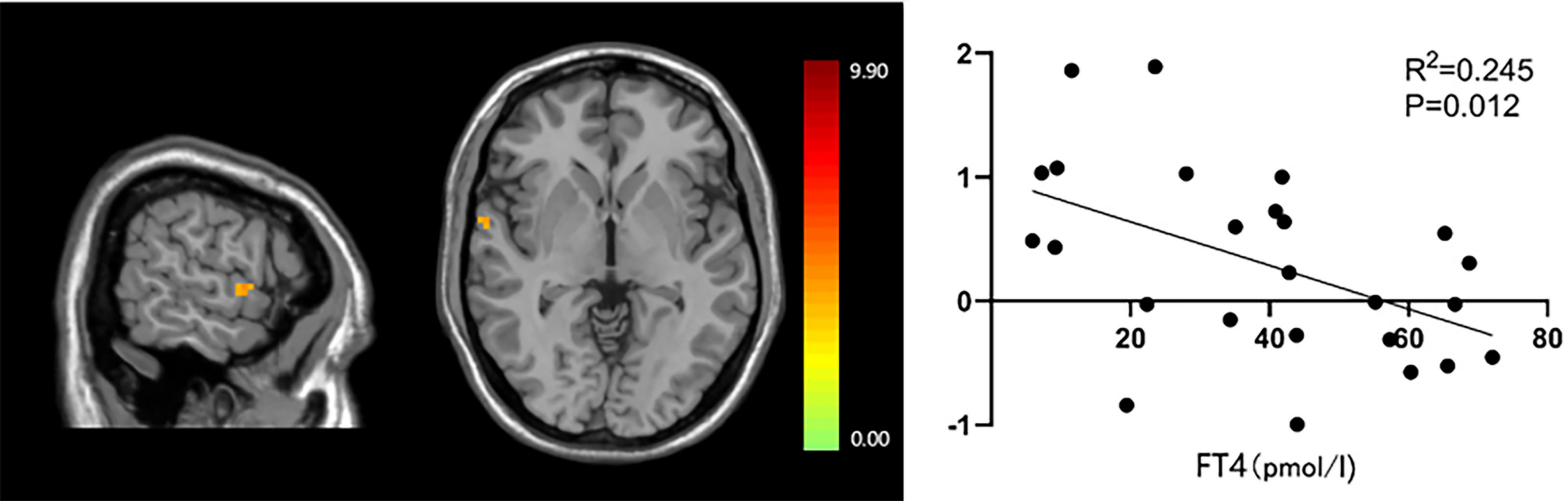
The significant correlation between functional connections in the right superior temporal gyrus and FT4 levels.

**Figure 4:**
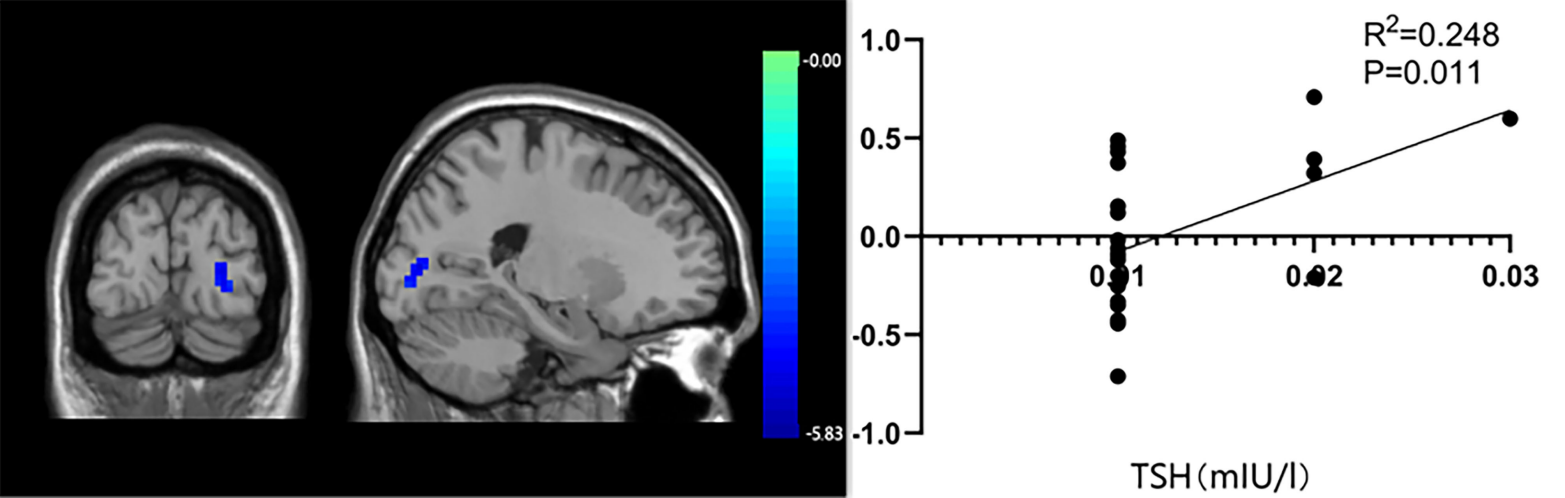
The significant correlation between occipital lobe functional connectivity and thyroid stimulating hormone levels.

The functional connection enhancement of SMN in the right superior temporal gyrus was significantly negatively correlated with FT4 level (P=0.012, r=-0.495) (**Figure 3**), and the weakened SMN functional connection in the occipital lobe was significantly positively correlated with TSH level (P=0.011, r =0.428) (**Figure 4**). The SMN and LFPN functional connectivity changes in other brain regions were not found to be significantly correlated with thyroid function parameters.

## Discussion

ATM is a rare but serious complication of hyperthyroidism. This disease has an insidious onset and is easily misdiagnosed and missed. It progresses rapidly and involves the muscles innervated by the medulla oblongata. Delayed diagnosis and treatment could be life-threatening. The symptoms of ATM have heterogeneity. Dysarthria and dysphagia are the most common symptoms (14), often accompanied by weakened strength of the pharyngeal muscle group. A small number of patients with ATM may experience language disorder, decreased computational power, hallucinations, et al., and even pyramidal tract and functional damage of corticobulbar tract (15). However, because the living brain histopathological specimens cannot be obtained at the onset of ATM patients, the current research on the mechanisms of ATM bulbar paralysis progresses slowly. A few scholars have found changes in brain functional areas in patients with thyroid disease (hyperthyroidism or hypothyroidism) with rs-fMRI. At present, there are few literatures about brain functional areas in ATM. The focus of our study is to use rs-fMRI technology to study whether there are changes in brain functional areas in ATM patients and further explore the possible pathogenesis of ATM. The use of this investigating tool is new and potentially important to help differentiate ATM from hyperthyroid patients without such complications.

ICA is a resting-state brain network data analysis method with good reliability and repeatability. Based on ICA, our study explored the difference between the functional connection of the SMN and the LFPN between the ATM group and nATM group and analyzed the relationship between functional connectivity and thyroid function parameters. Our data provided that the functional connections of both SMN and LFPN in the ATM group were changed, and the enhancement of SMN functional connectivity at the right superior temporal gyrus was significantly negatively correlated with FT4 level, and its weakening at the occipital lobe was significantly positively correlated with TSH level.

Dysphagia is one of the heterogeneous symptoms of bulbar paralysis in ATM patients. Swallowing function is one of the most complex somatic reflexes. Studies have shown that the cortical swallowing network involves several dispersed brain regions, including the primary sensorimotor cortex, insular lobe, frontal operculum, cingulate gyrus, temporal lobe, subcortical structure, cerebellum, etc. (16). These brain regions with tight functional connections regulate swallowing function together (17, 18). In our study, compared with the nATM group, the ATM group had functional connectivity changes in the precentral gyrus, postcentral gyrus, frontal operculum, cingulate gyrus, temporal lobe, and cerebellum etc. In the high-level cortical swallowing central network, the primary sensorimotor cortex (precentral gyrus is the cortical motor center and the postcentral gyrus is the cortical sensorium) is the most commonly excited area (19) and is involved in the swallowing-related muscle control and sensory feedback. Lin Zhicheng et al. (20) found that stroke patients with swallowing disorders had weakened functional connectivity in the precentral and postcentral gyrus, and the weakening degree was correlated with the severity of swallowing disorder, which is consistent with the results of our study. It is indicated that the primary sensorimotor cortex may be involved in and play an important role in the impairment of swallowing function of ATM patients. The cerebellum has long been considered critical for the control of motor (21, 22) and is a major target for thyroid hormone. The cerebellum integrates the received sensorimotor information and is involved in regulating the precision and coordination of swallowing-related muscle groups. We showed that the ATM group had functional connectivity changes in the anterior and posterior lobe of cerebellum compared with the nATM group. Although the sample size was small in our study, and there was no significant difference in FT4 levels between the two groups (p=0.08), the level of FT4 in the ATM group was higher than that in the nATM group. Therefore, we still speculated that the elevated thyroid hormone may affect the neurons in the cerebellum and led to ataxia in the swallowing-related muscles that it regulated. In addition, the cingulate gyrus, one of the core regions of the limbic system, has a high thyroid hormone receptor content and is very sensitive to changes in thyroid hormone levels. Excessive thyroid hormones tend to cause abnormalities in the structure and function of the limbic system, further leading to impaired swallowing initiation and oropharyngeal control function. Our data suggest the functional connection of the bilateral cingulate gyrus of the ATM group was changed, which further confirmed the importance of the cortical swallowing central network in the swallowing function of ATM patients. Excessive thyroid hormone secretion affected the normal activation of the cortical swallowing central network.

Dysarthria is another heterogeneous symptom of bulbar paralysis of ATM patients, which is mainly manifested by the degraded speech intelligibility caused by the dysfunction of the articulators, including the tongue, oral cavity, and pharynx. In recent years, modern brain imaging techniques have shown that speech production is not only controlled by specific speech centers (23) but also rely on the joint involvement of different brain regions, including the sensorimotor cortex, supplementary motor areas, inferior frontal gyrus, superior temporal gyrus, and cerebellum (24). They can form a complex speech function network to perform speech-related tasks. Further data from our group have shown that the ATM group had functional connectivity changes in the precentral gyrus, postcentral gyrus, cingulate gyrus, frontal lobe, right superior temporal gyrus, and cerebellum, compared with the nATM group. The frontal lobe is involved in several aspects of language such as semantic priming and processing, articulation and intonation. The temporal lobe, as an important hub of the language comprehension pathway for auditory information processing, forms a functional network for communication connection with the frontal lobe, which is located in the center of language processing (25). Lee et al. (26) applied rs-fMRI in their study and found that the anterior-ventral frontal lobe and posterior-lateral temporal lobe of Parkinson’s patients with dysarthria were associated with activation of cognitive resources during grammar and utterance processing. The rs-fMRI results in their study are similar to the results of the functional connectivity changes in the frontal and temporal lobes in our study. Taken together, these data suggested that the frontal and temporal lobes were jointly involved in the language execution and processing of ATM patients and the function of the motor speech center of ATM patients might be damaged. The cingulate gyrus is located in the supplementary motor area and is involved in complex emotional processes such as vocalization (27). Previous studies have shown that the local consistency of the left cingulate gyrus in patients with aphasia shows deactivation and a significant negative correlation with the values of the dysarthria scale, suggesting that dysarthria is associated with functional damage of the cingulate gyrus (28). Our study showed that the functional connectivity of SMN and LFPN of the ATM group were both enhanced at the cingulate gyrus, and it was speculated that the cingulate gyrus might try to play a functional compensatory role by enhancing functional connectivity during the dysarthria in ATM patients. In 2021, Ishani et al. (29) found cross-activation of the cerebral cortex and cerebellum during the execution of semantic and phonological association tasks in brain tumor patients with aphasia. The study of D’Mello et al. also suggested that the cerebellum was involved in phonological and semantic processing (30). The functional connectivity changes in the cerebellum of ATM patients were also observed in our findings. It was speculated that dysarthria in ATM patients might be related to functional connectivity changes in their cerebellums. In addition, it has been shown that damage of the bilateral parahippocampal gyrus is closely related to degraded speaking fluency (31). In our study, the ATM group had weakened functional connectivity in the bilateral parahippocampal gyrus, reflecting functional connectivity changes in the parahippocampal gyrus affected the modulation of language tasks in ATM patients.

In our study, the level of thyroid hormone in the ATM group was higher than that in the nATM group, but the difference was not statistically significant. Correlation analysis between thyroid hormone levels and brain regions with functional connectivity changes of the two groups showed that the higher the FT4 level, the weaker the SMN functional connectivity in the right superior temporal gyrus; the lower the TSH level, the less the weakening of SMN functional connectivity in the occipital lobe. Swallowing activity is a complex sensorimotor process involving sight, hearing, smell and taste at the same time. The occipital lobe is the visual cortical center, and the superior temporal gyrus is the primary auditory cortex; visual stimuli associated with swallowing activity need to be combined with auditory and audiovisual stimuli to activate brain regions related to swallowing activities (32). Therefore, we speculated that in some patients with hyperthyroidism, excessive thyroid hormone levels and feedback inhibition of TSH affected the functional connection of brain regions responsible for visual sense and auditory sense, impairing the compensatory function of ATM patients and affecting the swallowing function.

Our study has some limitations. First of all, the number in ATM group was small. In the future, observation with a large sample size will be conducted to deeply explore the characteristic effects of different courses of the disease, severity of clinical symptoms, treatment and other factors on the resting-state brain network. Secondly, it is expected that structural magnetic resonance will be combined with functional magnetic resonance imaging (fMRI) to explore the physiological mechanism of ATM patients in terms of structure and function.

In summary, based on ICA, we found characteristic changes in SMN and LFPN functional connectivity of ATM patients. Therefore, rs-fMRI can be applied to the research of the physiopathologic mechanism of ATM patients. It is expected to become one of the objective imaging indicators for early clinical intervention research on ATM patients.

## Data Availability Statement

The raw data supporting the conclusions of this article will be made available by the authors, without undue reservation.

## Ethics Statement

The studies involving human participants were reviewed and approved by Ethics Committee of the First Affiliated Hospital of Guangxi Medical University. The patients/participants provided their written informed consent to participate in this study.

## Author Contributions

YL and JZ contributed to the study conception and design. Data collection and analysis were performed by YL, ML, SH, XL, YQ, and ZL. The first draft of the manuscript was written by YL and ML. All authors commented on previous versions of the manuscript. JZ finalized the final manuscript. All authors read and approved the final manuscript.

## Funding

This study was supported by grants from the General Project of Guangxi Natural Science Foundation, China (2018GXNSFAA281141).

## Conflict of Interest

The authors declare that the research was conducted in the absence of any commercial or financial relationships that could be construed as a potential conflict of interest.

## Publisher’s Note

All claims expressed in this article are solely those of the authors and do not necessarily represent those of their affiliated organizations, or those of the publisher, the editors and the reviewers. Any product that may be evaluated in this article, or claim that may be made by its manufacturer, is not guaranteed or endorsed by the publisher.

## Abbreviations

ATM, acute thyrotoxic myopathy; FT3, Free triiodothyronine in serum; FT4, Free thyroxine in serum; ICA, independent component analysis; LFPN, left frontoparietal network; MNI, Montreal neurological institute; nATM: Non-acute thyrotoxic myopathy hyperthyroidism; RS-fMRI, resting-state functional magnetic resonance imaging; SMN, sensorimotor network; TSH, Thyroid stimulating hormone.
